# Mining Open Payments Data: Analysis of Industry Payments to Thoracic Surgeons From 2014-2016

**DOI:** 10.2196/11655

**Published:** 2018-11-30

**Authors:** Xu Na, Haihong Guo, Yu Zhang, Liu Shen, Sizhu Wu, Jiao Li

**Affiliations:** 1 Institute of Medical Information / Medical Library Chinese Academy of Medical Sciences & Peking Union Medical College Beijing China

**Keywords:** open payments data, pharmaceutical industry, thoracic surgeons, transfer of value

## Abstract

**Background:**

The financial relationship between physicians and industries has become a hotly debated issue globally. The Physician Payments Sunshine Act of the US Affordable Care Act (2010) promoted transparency of the transactions between industries and physicians by making remuneration data publicly accessible in the Open Payments Program database. Meanwhile, according to the World Health Organization, the majority of all noncommunicable disease deaths were caused by cardiovascular disease.

**Objective:**

This study aimed to investigate the distribution of non-research and non-ownership payments made to thoracic surgeons, to explore the regularity of financial relationships between industries and thoracic surgeons.

**Methods:**

Annual statistical data were obtained from the Open Payments Program general payment dataset from 2014-2016. We characterized the distribution of annual payments with single payment transactions greater than US $10,000, quantified the major expense categories (eg, Compensation, Consulting Fees, Travel and Lodging), and identified the 30 highest-paying industries. Moreover, we drew out the financial relations between industries to thoracic surgeons using chord diagram visualization.

**Results:**

The three highest categories with single payments greater than US $10,000 were Royalty or License, Compensation, and Consulting Fees. Payments related to Royalty or License transferred from only 5.38% of industries to 0.75% of surgeons with the highest median (US $13,753, $11,992, and $10,614 respectively) in 3-year period. In contrast, payments related to Food and Beverage transferred from 93.50% of industries to 98.48% of surgeons with the lowest median (US $28, $27, and $27). The top 30 highest-paying industries made up approximately 90% of the total payments (US $21,036,972, $23,304,996, and $28,116,336). Furthermore, just under 9% of surgeons received approximately 80% of the total payments in each of the 3 years. Specifically, the 100 highest cumulative payments, accounting for 52.69% of the total, transferred from 27 (6.05%) pharmaceutical industries to 86 (1.89%) thoracic surgeons from 2014-2016; 7 surgeons received payments greater than US $1,000,000; 12 surgeons received payments greater than US $400,000. The majority (90%) of these surgeons received tremendous value from only one industry.

**Conclusions:**

There exists a great discrepancy in the distribution of payments by categories. Royalty or License Fees, Compensation, and Consulting Fees are the primary transferring channels of single large payments. The massive transfer from industries to surgeons has a strong “apical dominance” and excludability. Further research should focus on discovering the fundamental driving factors for the strong concentration of certain medical devices and how these payments will affect the industry itself.

## Introduction

### Background

The term transfer of value means a direct or indirect transfer of value, whether in cash, in kind or otherwise, in connection with the development or sale of medicine [1]. Potential conflicts of interest arising from the transfer of value between the pharmaceutical industries and physicians could significantly affect clinical care, research findings, and physician decision-making. The risks and benefits of physician-industry financial relationships have long been hotly debated [2-4]. A recent investigation found a pattern of after-the-fact compensation by industry to those advising the US government on drug approvals [5].

Open health care–related data have been widely collected and analyzed [6,7]. The Physician Payments Sunshine Act of the US Patient Protection and Affordable Care Act (2010) was enacted in response to growing public interest and scrutiny regarding the financial relationship between physicians and the pharmaceutical and product industries [8,9]. The act mandates that drug and device manufacturers report individual payments of greater than US $10, or US $100 in aggregate annually, received by physicians and teaching hospitals. Physicians include doctors of Medicine, Osteopathy, Dentistry, Dental Surgery, Podiatry, Optometry, and Chiropractic Medicine who are legally authorized to practice. Industry reporting of financial remuneration for travel, gifts, and services rendered is now mandated by the US Centers for Medicare and Medicaid Services (CMS), and the resulting data are made publicly available through the Open Payments Program (OPP) database [10]. General payment records in the OPP provide the total value of general payments or other transfers of value to a particular recipient for a particular date. Each record includes identifying information for the physicians and teaching hospitals in the United States, identifying information for the applicable manufacturer, and applicable group purchasing organizations who made the payment, the total amount of payment, date of payment, nature of payment, associated drug or biological, etc [11].

According to the World Health Organization’s 2018 annual report [12], the majority of all noncommunicable disease deaths were caused by cardiovascular disease, accounting for 44% of 41 million deaths. The American Heart Association 2018 update on heart disease and stroke statistics has disclosed that approximately 92.1 million American adults are living with cardiovascular disease or the after-effects of stroke. Direct and indirect costs of cardiovascular diseases and stroke are estimated to total more than US $329.7 billion [13].

### Study Aims

Research has been conducted to quantify industry payments to different specialties by analyzing the OPP database [14-17]. However, compared with previous studies, we focused on analyzing the payment characteristics during a 3-year period and explored the regularity of the financial relationship between industries and thoracic surgeons.

## Methods

### Data Sources

We accessed the OPP general payment dataset, which is publicly accessible, from 2014-2016. We excluded the 2013 dataset due to incompleteness and inconsistencies in the OPP records, which may have led to deviation in reflecting the real situation. Our study focused on the non-research and non-ownership payments received by doctors of Thoracic Surgery. Therefore, we excluded payments for current or prospective ownership or investment interest and limited the physician specialty studied to Thoracic Surgery (Cardiothoracic Vascular Surgery). Payments valued at US $0 were also excluded. There are a few industries with the same name and different ID numbers in the OPP database; however, almost all annual cumulative payment amounts for the different IDs were either less than US $1000, or the payment amounts were no more than US $10, and the majority were transferred to the same surgeon or were associated with the same drug or biological agent. Therefore, we counted the number of industries with distinct names. Our final cohort included 197,592 payments from 446 industries to 4552 surgeons in 3 years.

### Payment Categories

The CMS has defined 16 categories for the nature of payment. The six major expense categories involved in this study included the following: Consulting Fees; Compensation for services other than consulting, including serving as faculty or as a speaker at a venue other than a continuing education program (abbreviated as “Compensation”); Travel and Lodging; Food and Beverage; Royalty or License; and Education (see Multimedia Appendix 1). These six categories were selected because they account for greater than 95% of total payments in the database.

### Statistical Analysis

All analyses were performed with R 3.4.1. Due to the significant skew of the data, the results are presented as median payments with interquartile ranges (IQR), and a log transformation was performed on payments prior to graphing boxplots to enable visual clarity. Descriptive statistics were calculated to analyze the distribution of annual payments with a single payment greater than US $10,000, payments by major expense categories, and payments by the 30 highest-paying industries. A chord diagram was used to show the distribution of the 100 highest cumulative payments from pharmaceutical industries to thoracic surgeons in the 3-year period.

## Results

### Distribution of Annual Payments

There were 61,963 payments totaling US $21,036,972 transferred from 299 industries to 3667 thoracic surgeons in 2014; 64,558 payments totaling US $23,304,996 transferred from 282 industries to 3613 surgeons in 2015; and 71,071 payments totaling US $28,116,336 transferred from 283 industries to 3717 surgeons in 2016. The registered number for Thoracic Surgery (Cardiothoracic Vascular Surgery) in the National Provider Identifier (NPI) Database was 5614, excluding medical groups [18]. The NPI number is a unique 10-digit number issued by the CMS to health care providers in the United States. Based on the NPI, 81.08% of the registered surgeons received a transfer of value in the 3 years; furthermore, two-thirds of the surgeons received a payment each year. The distribution of payments to thoracic surgeons significantly skewed toward smaller payments over the 3 years. The median dollar amount (IQR) of the annual payments were 44 (17-125), 42 (17-123), and 44 (17-126). Furthermore, the proportion of maximum payment increased dramatically from 5.58% (US $1,173,913), to 10.95% (US $2,551,007), to 17.78% (US $5,000,000) of the total. The result illustrated that the skewed distribution kept expanding overall from 2014-2016.

### Distribution by Six Major Expense Categories

**Figure 1:**
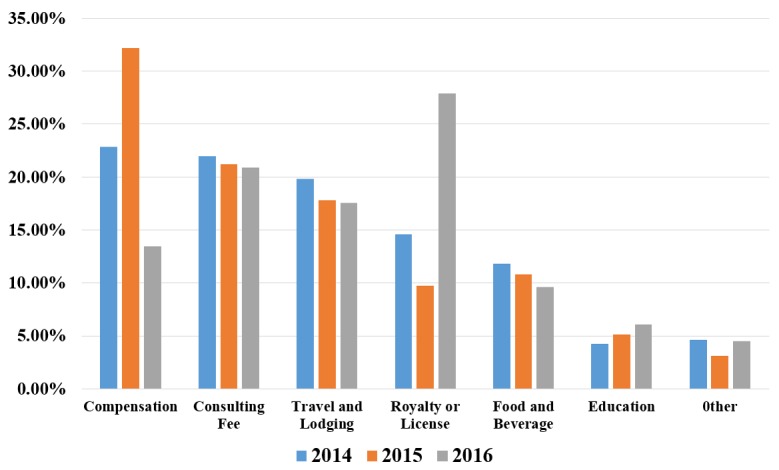
Distribution of pharmaceutical industries payments to thoracic surgeons by major categories, 2014-2016.

Six major expense categories accounted for 95.34%, 96.91%, and 95.53% of all payments in 2014, 2015, and 2016, respectively. The most common category associated with payments to thoracic surgeons was Compensation, accounting for 22.84% (US $4,804,701) and 32.18% (US $7,499,516) of all payments in 2014 and 2015, respectively. However, the most common payment changed to Royalty or License (US $7,846,886), which received 27.91% of the total in 2016. The second largest share of payments was Consulting Fees, accounting for 21.95%, 21.23%, and 20.91%, followed by Travel and Lodging 19.84%, 17.81%, and 17.54% (see Figure 1 and Multimedia Appendix 2). In addition, the proportion of Consulting Fees, Travel and Lodging, and Food and Beverage decreased gradually, whereas payments in the Education category increased by comparison.

**Figure 2:**
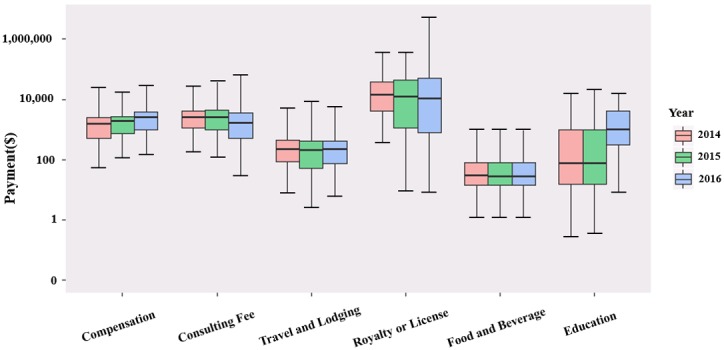
Boxplot of pharmaceutical industries payments to thoracic surgeons by six major categories, 2014-2016 (log transformation performed on payments prior to graphing boxplots for visual clarity).

**Figure 3:**
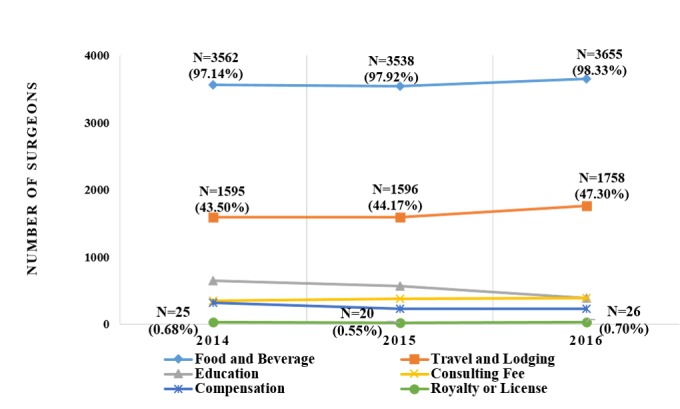
Distribution of pharmaceutical industries payments to thoracic surgeons by number of surgeons, 2014-2016.

The payment category with the highest median was Royalty or License. The median dollar payment (IQR) for this category and the percentage of surgeons receiving them were US $13,753 (4122-38,260) to 0.68%, US $11,992 (1132-43,054) to 0.55%, and US $10,614 (766-50,000) to 0.70%. In contrast, the payment category with the lowest median was Food and Beverage, in which the median payments in dollars (IQR) were US $28 (15-80) to 97.14%, US $27 (15-79) to 97.92%, and US $27 (14-77) to 98.33% (see Figures 2 and 3). In addition, the median of the Compensation and Education categories increased annually, while the median of the Royalty or License, and Consulting Fees categories decreased continuously.

Single payments greater than US $10,000 accounted for 35.19%, 39.44%, and 41.16% of the total during the 3 years, which transferred from 14.05% of the industries to 2.35% of the surgeons; from 13.83% of the industries to 2.13% of the surgeons; and from 11.66% to 2.02% of the surgeons, respectively (Table 1). The proportion of single payments greater than US $10,000 increased substantially; in contrast, the percentage of surgeons and industries decreased gradually. Royalty or License, Compensation, and Consulting Fees took up the three highest proportions in the 3 years. Moreover, 97.40%, 96.92%, and 98.95% of payments in the Royalty or License category were greater than US $10,000 from 2014-2016; 45.96%, 63.89%, and 30.29% of payments in the Compensation category were greater than US $10,000; and 36.11%, 35.21%, and 35.91% of payments in the Consulting Fees category were greater than US $10,000. Additionally, 8.62%, 8.36%, and 8.77% of surgeons with annual cumulative payment amounts greater than US $10,000 received 78.42% (US $16,496,272), 80.63% (US $18,790,319), and 81.82% (US $23,004,994), respectively.

### Distribution by 30 Highest-Paying Industries and 100 Highest Cumulative Payments

**Figure 4:**
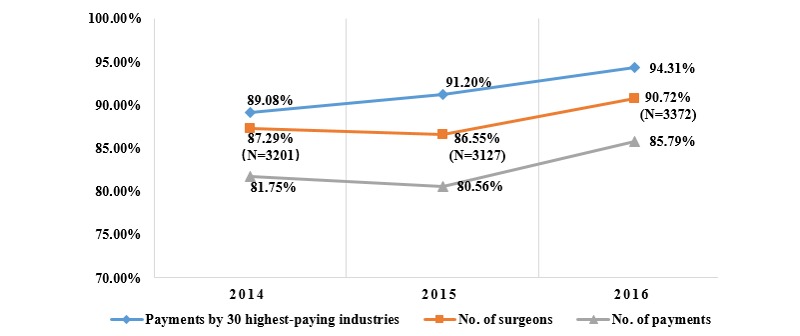
Distribution of 30 highest-paying pharmaceutical industries, 2014-2016.

The 30 highest-paying industries made up the vast majority (89.08%) of the total transferred to 3201 (87.29%) surgeons through 50,656 (81.75%) payments, 91.20% of the total transferred to 3127 surgeons through 52,010 payments, and 94.31% of the total transferred to 3372 surgeons through 60,975 payments during the 3 years (see Figure 4).

**Figure 5:**
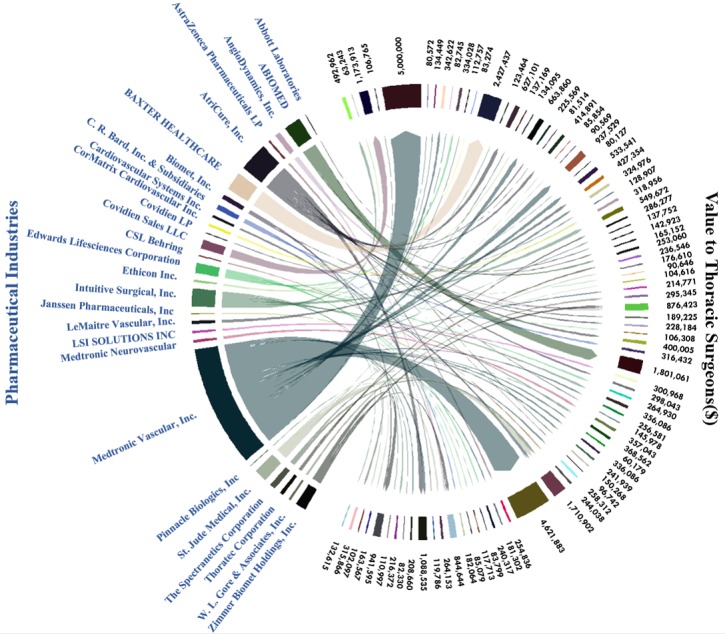
Financial relationships of the 100 highest cumulative payments from pharmaceutical industries (N=27) to thoracic surgeons (N=86) in 3 years.

The 100 highest cumulative payments accounted for 52.69% (US $38,179,324) of the total transferred from 6.05% of the pharmaceutical industries to 1.89% of the thoracic surgeons in 3 years. The five highest-paying industries were Medtronic Vascular, Inc. (41.00% of the 100 highest cumulative payments), AtriCure, Inc. (9.85%), Baxter (6.36%), Intuitive Surgical, Inc. (6.14%), and Abiomed (5.77%). Moreover, 7 surgeons received payments greater than US $1,000,000; 12 surgeons received payments greater than US $400,000; and 68 surgeons received payments greater than US $80,000. Only 8 surgeons received a transfer of value from more than one industry (see Figure 5).

**Table 1:** Distribution of payments with single payments greater than US $10,000 by major categories, 2014-2016^a^.

Year and Category		Royalty or License	Compensation	Consulting Fee	Travel and Lodging	Education	Other	Total
**2014**								
	Surgeons, n	16	17	37	21	2	N/A^b^	86 (2.35)^c^
	Industries, n	10	9	25	11	2	N/A	42 (14.05)^c^
	Payments, US $ (%)	2,992,270 (97.40)	2,208,319 (45.96)	1,667,119 (36.11)	162,183 (3.89)	27,000 (3.00)	345,129 (N/A)	7,402,020 (35.19)
	Payments, n	51	20	62	14	2	26	175
**2015**								
	Surgeons, n	13	19	33	12	3	N/A	77 (2.13)^c^
	Industries, n	9	8	18	8	2	N/A	39 (13.83)^c^
	Payments, US $ (%)	2,197,409 (96.92)	4,791,506 (63.89)	1,741,916 (35.21)	214,015 (5.16)	60,500 (5.04)	185,200 (N/A)	9,190,546 (39.44)
	Payments, n	41	36	72	17	4	15	190
**2016**								
	Surgeons, n	16	21	32	17	1	N/A	75 (2.02)^c^
	Industries, n	10	3	20	6	1	N/A	33 (11.66)^c^
	Payments, US $ (%)	7,764,333 (98.95)	1,146,097 (30.29)	2,111,663 (35.91)	351,156 (7.12)	15,000 (0.87)	184,093 (N/A)	11,572,342 (41.16)
	Payments, n	41	52	53	26	1	14	187

^a^The percentage of payments for major categories all mean the proportion of payments with a single payment greater than $10,000 of the total payments for the category (see Multimedia Appendix 2).

^b^N/A: not applicable.

^c^Value is reported as n (%).

## Discussion

### Principal Findings

The number of surgeons and industries was stable, while the total annual payments and the number of payments grew steadily from 2014-2016. Our study showed that just under 9% of surgeons with annual cumulative payment amounts greater than US $10,000 received approximately four-fifths of the annual payments in 3 consecutive years, indicating that the distribution of payments to thoracic surgeons appears to have a remarkable skewness. The result was quite consistent with those from related studies [19,20]. We also found that the industries are inclined to transfer tremendous value to surgeons in a single payment (great than US $10,000). This trend has gradually expanded, with the rapid growth of the proportion of payments of single payments greater than US $10,000 and three-quarters of payments less than US $130 in the 3 years. There were significant differences in the categories of the industry payments (Table 2). The six most common payments accounted for more than 95% of all payments during the 3 years. This finding indicated that the unevenly distributed payments are highly concentrated in certain categories. The extremely unbalanced payment distribution by categories in Thoracic Surgery is quite similar to those of other specialties, although there exists variation in different specialties [21-24].

By comparison, we realized that the three highest-paying categories with single payments greater than US $10,000 were Royalty or License, Compensation, and Consulting Fees. This finding revealed that these categories are the most primary transferring channels of major values from industries to surgeons. Moreover, the distribution of these payments showed a remarkable skewness. In our study, Royalty or License category was transferred from the lowest percentage (5.38%) of industries to 0.75% of the surgeons, of which single payments greater than US $10,000 accounted for 98.24% in the 3-year period and were based on physicians’ intellectual property. Similarly, approximately half of the Compensation category and one-third of the Consulting Fees category were transferred to less than 1% of the surgeons, with single payments greater than US $10,000, requiring expertise on a medical product or treatment as well as surgeon participation as faculty or a speaker for noncontinuing education.

**Table 2:** Payment characteristics by major categories in the 3-year period^a^.

Payment category	Payments, %	Industry coverage, %	Surgeon coverage, %	Single payment >US $10,000, %	Professional skills requirements^b^
Royalty or license	18.20	5.38	0.75	98.24	Physician’s intellectual property
Consulting fee	21.31	30.04	14.94	35.75	Advice on medical product or treatment
Compensation	22.20	13.90	10.96	50.63	Speaking, training, and noncontinuing education
Travel and lodging	18.30	39.01	57.62	5.49	None
Food and beverage	10.64	93.50	98.48	None	None
Education	5.26	17.94	27.39	2.69	Imparting or acquiring of particular knowledge or skills

^a^The percentage of payments, industry coverage, surgeon coverage, and single payment >US $10,000 all refer to the cumulative percentage in the 3-year period.

^b^Professional skills requirements are based on the Centers for Medicare and Medicaid Services’ definitions of six major expense categories.

One plausible reason for these results is that the monopoly of the patent market leads to a tiny proportion of industries that occupy vast market shares; hence, more emphasis is being placed on technological innovation and development to maintain superiority. To do this quickly, industries may seek out leading authorities on thoracic surgeons as cooperative partners, who, due to their deeper scientific and medical experience, are more likely to make patents of inventions in Thoracic Surgery. Industries could establish a long-term partnership, obtain the technology license, and sell their highly profitable patented products on the market by paying large-value Royalty or License, Compensation, and Consulting Fees to these surgeons. In another aspect, related research warned that enormous payments from a few major industries to certain surgeons who advised the US Food and Drug Administration on the approval for the industries’ new drugs fit a pattern of what might be called pay-later conflicts of interest [5].

Characteristics of Food and Beverage payments are in sharp contrast to those of Royalty or License. In fact, 10.64% of payments in this category, with 75% of payments less than US $80, transferred to more than 98% of the surgeons from over 93% of the industries in 3 consecutive years, which was consistent with the findings from multiple related studies [25-27]. These results imply that the transfer of value from industries to surgeons in this category was much more widespread in comparison to others. Additionally, this payment has few connections with surgeons’ clinical or professional skills. The outcome might be explained by the fact that patients may have a more negative view on payments of food in related studies [28,29]. Similarly, nearly one-fifth of the payments from 39.01% of the industries transferred to 57.62% of the surgeons through the Travel and Lodging category, with 75% of payments less than US $450 in 3 consecutive years; this category is also unrelated to professional skills. It is highly noteworthy that approximately 30% of the total transfer of value was widely transferred to surgeons with no requirement of professional knowledge or skills. Related research found that the receipt of industry-sponsored meals was associated with an increased rate of prescribing the brand-name medication that was being promoted [30]. Pharmaceutical industries provide hundreds of millions of dollars to physicians for food and beverages with the expectations of good returns [31]. Therefore, we are deeply convinced that these sizable payments have quite a widespread impact on industries’ product promotion to surgeons.

In another aspect, it should be noted that total payments in the Education category grew remarkably. Furthermore, the number of industries and surgeons reduced significantly over the 3 years, indicating that payment tendency in the Education category was inclined to a highly centralized model. The major reason for this result is that there were substantial imbalances of payments among industries, with the highest-paying industry accounting for 67.94% (US $610,789) in 2014, 77.96% (US $936,205) in 2015, and 94.65% (US $1,622,728) in 2016. Our research found that the largely uneven distribution of industry payments also exists in other expense categories. Therefore, we can confirm that the payment growth is driven mainly by the few highest-paying industries.

One of the most interesting findings in our study is that the proportion of payments from the 30 highest-paying industries was approximately nine out of ten over 3 years, consistently accounting for only one-tenth of total industries. It is noteworthy that the payment amount is highly correlated with the size of the industries. The majority of 30 highest-paying industries were in the list of the world’s top 20 leading medical device and diagnostic companies in 2016 based on Evaluate MedTech’s annual report [32]. The vast majority of these payments only transferred to less than 9% of surgeons, accordingly. Moreover, 7 surgeons (0.15%) received more than US $1,000,000 in 3-year period. A large share of all payments was skewed toward a small fraction of top earners; this pattern has also been observed in other specialties [23,33,34]. Based on the above results, we tentatively suggest that there exists the famous “apical dominance” in the transfer of value. This effect was described as the control exerted by the terminal bud over the outgrowth of lateral buds in plant physiology, which is also a widespread phenomenon in economics. Due to the extremely uneven development, tremendous transfers are highly concentrated on the dominant industries and leading surgeons.

Additionally, the 100 highest cumulative payments, accounting for more than half of the total, transferred from a tiny percentage of industries to a minority of surgeons. Over 90% of these surgeons received hundreds of thousands of dollars from only one specific industry during the 3 years. These results strongly indicate that leading surgeons have a long-term exclusive partnership with dominant industries, particularly for large payment amounts. On the one hand, Royalty or License could be transferring to an individual industry due to its monopoly and exclusivity. On the other hand, key partners might understand the industries’ commercial secrets through consultations and other forms of cooperation. Therefore, industries do not wish for them to cooperate with competitors in the current and fierce business competition.

### Limitations

There are several limitations to this study. First, it should be noted that this study has examined only non-research and non-ownership payments, which may lead to deviations in reflecting the real situation. Second, the database relies on accurate reports from manufacturers. There may be inaccurate attribution of payments into categories, to individuals, or to affiliated institutions by reporting companies in the database. The CMS redacted more than 40% of all reported payment records in the first year. Finally, our research mainly focuses on the payment characteristics of six major expense categories, which may not completely reflect all data features.

### Conclusions

There exists a great discrepancy in the distribution of payments by categories. Royalty or License, Compensation, and Consulting Fees are the primary transferring channels of single large payments. The massive transfer from industries to surgeons has a strong “apical dominance” and excludability. Furthermore, our study provides evidence that payments by the 30 highest-payment manufacturers were specifically targeting certain medical devices during the 3 years. Further research should focus on discovering the fundamental driving factors for the strong concentration of certain medical devices and how these payments will affect the industry itself.

## Appendix

Multimedia Appendix 1 The CMS definitions of six major expense categories.

Multimedia Appendix 2 Annual payments distribution of payments from industries to surgeons, 2014-2016.

## Abbreviations

CMS: Centers for Medicare and Medicaid Services
IQR: interquartile ranges
NPI: National Provider Identifier
OPP: Open Payments Program
