# Supplementary material for: Mining Open Payments Data: Analysis of Industry Payments to Thoracic Surgeons From 2014-2016
Source: J Med Internet Res. 2018 Nov 30;20(11):e11655. doi: 10.2196/11655 (PMC6294877; doi:10.2196/11655)
Supplement: Multimedia Appendix 1 [file jmir_v20i11e11655_app1.pdf]

Multimedia Appendix 1. The CMS definitions of six major expense categories.

Nature of payment	Definition
Consulting fee	Payments made to physicians for advice and expertise on a particular medical product or treatment, typically provided under a written agreement and in response to a particular business need. These payments often vary depending on the experience of the physician being consulted.
Compensation for services other than consulting, including serving as faculty or as a speaker at an event other than a continuing education program.	Payments made to physicians for speaking, training, and education engagements that are not for continuing education.
Food and beverage	Food and beverage.
Travel and lodging	Travel and lodging
Royalty or license	Royalty or other payment based on sales of products that use a physician's intellectual property.
Education	This category generally includes payments or transfers of value for classes, activities, programs or events that involve the imparting or acquiring of particular knowledge or skills, such as those used for a profession. This category can include things like textbooks and medical journal articles.

[a] Source: https://www.cms.gov/openpayments/about/natures-of-payment.html
