# Supplementary material for: Mining Open Payments Data: Analysis of Industry Payments to Thoracic Surgeons From 2014-2016
Source: J Med Internet Res. 2018 Nov 30;20(11):e11655. doi: 10.2196/11655 (PMC6294877; doi:10.2196/11655)
Supplement: Multimedia Appendix 2 [file jmir_v20i11e11655_app2.pdf]

Multimedia Appendix 2. Annual payments distribution of payments from industries to surgeons, 2014-2016.

Payment type	2014				2015				2016			
	Surgeons	Industries	Total, Dollars	Median, Dollars (IQR)	Surgeons	Industries	Total, Dollars	Median, Dollars (IQR)	Surgeons	Industries	Total, Dollars	Median, Dollars (IQR)
Compensation	319	45	4,804,701	1,500 (500-2,500)	231	49	7,499,516	1,832 (750-2,748)	228	39	3,783,388	2,500 (1,000-3,750)
Consulting Fee	352	88	4,616,707	2,500 (1,093-4,000)	382	77	4,947,661	2,500 (1,000-4,477)	389	76	5,879,663	1,600 (500-3,570)
Travel and Lodging	1595	118	4,174,324	219 (88-450)	1596	110	4,151,334	199 (52-401)	1758	102	4,930,788	219 (73-411)
Royalty or License	25	15	3,072,302	13,753 (4,122-38,260)	20	14	2,267,285	11,992 (1,132-43,054)	26	17	7,846,886	10,614 (766- 50,000)
Food and Beverage	3562	278	2,490,433	28 (15-80)	3538	262	2,517,923	27 (15-79)	3655	263	2,704,603	27 (14-77)
Education	644	60	899,064	75 (15-1,000)	563	41	1,200,899	75 (15-1,000)	387	37	1,714,406	1,000 (300-4,000)
Other	N/A[a]	N/A	979,441	N/A	N/A	N/A	720,378	N/A	N/A	N/A	1,256,602	N/A
Total	3667	299	21,036,972	44 (17-125)	3613	282	23,304,996	42 (17-123)	3717	283	28,116,336	44 (17-126)

[a]N/A: not applicable.
